# Monthly Summary

**Published:** 1897-01

**Authors:** 


### Monthly Summary.

FORMALIN IN DENTAL PRACTICE.—Abraham is quoted as follows:—By the use of this substance he has been enabled to avoid that form of secondary periostitis that so often complicates the treatment of periodontitis. It is his belief that the vapors of formalin have a regenerative influence upon the diseased periodontium and promote *restitutio ad integram.* The method of formalin treatment made use of by the author more recently is less complicated, and less likely to cause pain by tha formalin accidentally overflowing on the surrounding tissue. He makes use of a powder calc. sulphas., 200; hydrarg. bichlor., 4. These to be finely mixed; also a liquid form, acid sulphuric, 32; formalin, 100; aq. distil., 100. A few drops of the liquid are rubbed on a glass plate together with sufficient of the powder to form a paste, which, introduced into the previously dried root-canal, solidifies in a few minutes. The crown can be filled with any stopping desired. The small quantity of corrosive sublimate and sulphuric acid in the paste does not have any discoloring or corrosive effect on the tooth. The above proportions have been found to be the best as a result of numerous trials, and this formalin root-cement may be employed safely after coutery of the pulp, etc., and for closing the foramen piacale, In a few cases, after employing this cement a slight periostitis without pain has appeared, continuing only one or two days.—*Zahnarztl'ches Wochrnblatt.*

---

My theory for several years has been, in reference to obtunding sensitive dentine, that the best method is to have clean, sharp burs, steady hands, an engine revolving at a moderate rate of speed, but running steadily, telling the patient you are going to hurt him a little, and then doing the work deftly and quickly. Up to the present time I have not found anything that I would trade off for my sharp, up-to-date instruments, deftly handled.—Dr. Osmon, in *International Dental Journal.*

CATAPHORESIS SIMPLY EXPLAINED.—You can now have a tooth pulled painlessly, and without gas, by electricity. Instantaneous? Why of course; but the point is that there is no pain. You need not bother with nitrous-oxide gas, which is very distressing in its effects upon some people. The up-to-date American dental surgeon has a very simple apparatus, which consists most importantly of a battery not much bigger than a cigar box. The battery has a queer little attachment called a "vibrator," which is nothing more or less than a short strip of metal fastened at both ends. When the current is on the strip vibrates at such a rate of speed that it hums. The operator tunes it up with a tuning fork until it gives out the note A; then he knows that the current is just right. The person in the dentist's chair grasps two handles which are connected with the battery by wires. At the same time the operator seizes his forceps which likewise are on the end of a wire proceeding from the battery. He touches the tooth, completing the circuit, and instantly a local anesthesia is produced. The tooth and neighboring gum are rendered for the moment insensible to pain, and out comes the offending molar or incisor with a dexterous twist. Before the patient has had time to realize what has happened he is gleefully paying the fee.—*S. F. and Pacific Druggist.*

---

IN preparing a tooth for the reception of a porcelain crown (Logan or Richmond), before excising the natural crown, if you will take a piece of French rubber tubing, about one-eighth inch wide and a little smaller than the tooth to be crowned, carefully work it up on the neck of the tooth and as close to the gum as you can get without causing too much pain, allowing the patient to wear it for forty-eight hours, you can then face the root off under the gum line without laceration, hemorrhage or discomfort to your patient, which I consider quite an advantage in doing a nice piece of crown-work.

If natural crown is broken off build down with cement sufficient to give room to adjust rubber tube.—Dr. F. E. Judson, "Ohio Dental Journal."

DR. JACK'S VIEWS ON IMPLANTATION.—It is unfortunate that in too many instances planted teeth, which have become fixed and useful organs, have, after several years, from causes not entirely clear, yielded to absorption and have become useless. The termination of these cases seem to come with suddenness and without warning of the resorptive process. The cause of this result we have to look for in a depressed trophic state of the environment, in which condition it is not unreasonable to expect tissue of repair to be the first to yield. In any defective condition of the nutrition of a part this result is in consonance with well-fixed principles.

We have also to consider that the alveolar process is a provisional structure, and that its tendency is, on the occurrence of irritation or of impaired nutrition or by static conditions, to yield to resorption. The fact that it is provisional would impress on it the tendency to this structural change.

Notwithstanding the prognosis of planted teeth is at present precarious the promise of useful operations is so large, when selection is made of subjects in sound health and without cachetic taints, that, where plantation is required for the good of the patient, it should be performed. The probabilities are that greater experience will lead to improved methods and more certain results.—*Cosmos.*

---

Dr. J. W. Cormany: While we are on the subject of hypnotism I am going to give my experience of it. While I was preparing a cavity for a patient and had nearly completed the work, I suggested that it would hurt a little, and as quick as a flash she flew up and said, "What did you tell me that for. You have not hurt me a particle up to this time." She was a christian scientist and had hypnotized herself up to this time and I spoiled it all.

Dr. Newkirk: Dr. Cormany's little story reminds me of an experience of my own, which fully convinced me of the value of faith cure and power of the imagination. I had a patient two or three years ago, a lady who had made a study of this matter, and at the close of a series of operations she told me that she had been treating herself by faith cure, or mental processes, while the operations were going on, and that whereas she had formerly suffered a great deal, this time she had not suffered at all. I will say further for this lady that she is thoroughly consistent in her logic. She carries the applications of her principles into business matters. She imagined she had suffered no pain; she also imagines now that her bill is paid. (Laughter.)—*Dental Review*.

---

THE USE OF ROENTGEN RAYS.—The man Montague who swallowed his denture while in prison at Dundee, has at last got rid of his unwelcome intruder. His stomach was photographed by Roentgen light on an 18 by 14 inch plate, with an exposure of one and a half hours. A faint negative was the result, showing something like a set of teeth above the stomach. It was blurred and in duplicate, but this was supposed to be due to the man's motion in breathing, and also to the plate having shifted its position. A careful examination of the stomach was performed with no result, but the plate was afterwards found in the intestine and released. We wonder if the faint image on the negative was that of the teeth, or whether the denture had again changed its position, as the operation was performed twenty-four hours after the photograph was taken. In the case of a vulcanite denture only the teeth and clasps would cast a shadow, the plate being transparent to the rays.—"British Journal of Dental Science."

---

ADAM is possibly the only man that never suffered from having a tooth pulled, and I expect he is the only one who has missed that delightful process, for somehow or other the Almighty arranged that for him so that no dentist was needed and whether from Adam's time science has grown any less, or whether it has increased as the years have gone by, necessitating a special art and a special science for the alleviation of the woes of mankind, we cannot say, but we do know that American dentistry is rapidly taking its place among the progressive and polite sciences of the world.—F. V. Loos, in *Western Dental Journal*.

SMOKING AND INTELLECTUAL LABOR.—Dr. Drysdale, writing to the British Medical Journal, apropos of certain recently published statistics of smokers among the students of American colleges, recalls some facts discovered by Bertillon in 1855. He found on inquiry made by him concerning the pupils of the Polytechnique School of Paris that 108 of the pupils smoked and 52 did not smoke. He then arranged the 160 pupils into eight divisions, according to the places they held in examination, 20 in each rank, and found of the 20 who stood highest 6 were smokers and 14 non-smokers. Of the next 20, 10 were smokers and 10 non-smokers; of the next 20, 11 smoked and 9 did not smoke; thus showing how much higher the non-smokers stood intellectually than the habitual smokers. He also found that the mean rank of the smoker, as compared with that of the non-smoker, deteriorated from their entering to their leaving the school. As a result of Bertillon's the minister of public instruction of France issued a circular addressed to the director of schools and colleges, forbidding the use of tobacco and cigars to students.—*Medical Record.*

---

FOREIGN DENTISTS IN HUNGARY.—It has for a long time been a disputed question whether dentists who have obtained dental diplomas abroad should be permitted to practice in Hungary. Considering that only qualified medical men are allowed to practice dentistry in Hungary, and that there is no special examining board for dentistry in this country, the government has declared that for the future a license in another country will be valid only if its possessor is a qualified medical man who acquired his diploma of M. D. in one of those universities which are recognized in Hungary.—*The Lancet.*

---

TEMPORARY STOPPING.—Modeling composition forms an excellent temporary stopping. It is a non conductor, and the temperature of the mouth keeps it in a condition to be easily removed. It will wear for weeks.—G. V. N. Relyea, in *Dominion Journal.*

